# Rare missense substitutions in the mitochondrial DNA genes in patients with ventricular tachycardia

**DOI:** 10.18699/vjgb-25-74

**Published:** 2025-09

**Authors:** M.V. Golubenko, N.P. Babushkina, V.A. Korepanov, N.R. Valiakhmetov, T.A. Atabekov, K.N. Vitt, A.A. Zarubin, O.A. Makeeva, S.A. Afanasiev, R.E. Batalov, A.A. Garganeeva, M.S. Nazarenko, V.P. Puzyrev

**Affiliations:** Research Institute of Medical Genetics, Tomsk National Research Medical Center of the Russian Academy of Sciences, Tomsk, Russia; Research Institute of Medical Genetics, Tomsk National Research Medical Center of the Russian Academy of Sciences, Tomsk, Russia; Cardiology Research Institute, Tomsk National Research Medical Center of the Russian Academy of Sciences, Tomsk, Russia; Research Institute of Medical Genetics, Tomsk National Research Medical Center of the Russian Academy of Sciences, Tomsk, Russia; Cardiology Research Institute, Tomsk National Research Medical Center of the Russian Academy of Sciences, Tomsk, Russia; Cardiology Research Institute, Tomsk National Research Medical Center of the Russian Academy of Sciences, Tomsk, Russia; Research Institute of Medical Genetics, Tomsk National Research Medical Center of the Russian Academy of Sciences, Tomsk, Russia; Research Institute of Medical Genetics, Tomsk National Research Medical Center of the Russian Academy of Sciences, Tomsk, Russia; Cardiology Research Institute, Tomsk National Research Medical Center of the Russian Academy of Sciences, Tomsk, Russia; Cardiology Research Institute, Tomsk National Research Medical Center of the Russian Academy of Sciences, Tomsk, Russia; Cardiology Research Institute, Tomsk National Research Medical Center of the Russian Academy of Sciences, Tomsk, Russia; Research Institute of Medical Genetics, Tomsk National Research Medical Center of the Russian Academy of Sciences, Tomsk, Russia; Research Institute of Medical Genetics, Tomsk National Research Medical Center of the Russian Academy of Sciences, Tomsk, Russia

**Keywords:** mitochondrial DNA, heart arrhythmia, ventricular tachycardia, missense substitutions effects, genetic variant pathogenicity assessment, митохондриальная ДНК, аритмия, желудочковая тахикардия, эффект миссенс-замен, оценка патогенности генетических вариантов

## Abstract

Human mitochondrial DNA (mtDNA) exhibits high population-level polymorphism. While certain pathogenic mtDNA variants are known to cause hereditary mitochondrial syndromes, often presenting with cardiac arrhythmias, life-threatening ventricular tachycardia (VT) itself is a major risk factor for sudden death in cardiovascular diseases. The aim of the work was to study rare ("private") missense substitutions in the mtDNA of patients with documented episodes of ventricular tachycardia in comparison with patients with ischemic heart disease without life-threatening heart arrhythmias and individuals without clinical manifestations of cardiovascular diseases. The sequencing of mtDNA was performed using high-throughput sequencing methods. Specialized algorithms predicting the effect of gene variants were used to assess the effect of missense substitutions. Comparative analysis of the spectrum of the identified amino acid substitutions in the studied groups showed that about 40 % of the individuals in all three groups were carriers of "private" missense variants in mtDNA. However, among such substitutions, the variants classified by the APOGEE2 predictor as "variants of uncertain significance" (VUS) were more common in the group of patients with heart arrhythmias than in the control group, where "private" missense substitutions of the VUS category were not detected (p = 0.0063 for Fisher’s exact test). In addition, the groups differed in their phred-ranked Combined Annotation Dependent Depletion (CADD) scores, which were lower for individuals in the control group. The results indicate that rare mtDNA variants may contribute to predisposition to cardiovascular disease – in particular, to the risk of developing ventricular tachycardia by some patients.

## Abstract (ru)

Митохондриальная ДНК (мтДНК) характеризуется высоким полиморфизмом в популяциях. При этом некоторые патогенные варианты мтДНК могут приводить к развитию наследственных митохондриальных синдромов, симптоматика которых включает в том числе нарушения ритма сердца. С другой стороны, жизнеугрожающие аритмии, в виде желудочковой тахикардии, являются фактором риска внезапной смерти у пациентов с сердечно-сосудистыми заболеваниями. Целью работы стало исследование редких («приватных») миссенс-замен в мтДНК пациентов с зарегистрированными эпизодами желудочковой тахикардии в анамнезе в сравнении с пациентами с ишемической болезнью сердца без жизнеугрожающих нарушений ритма и индивидами без клинических проявлений сердечно-сосудистых заболеваний. Определение последовательности мтДНК проводили с помощью методов высокопроизводительного секвенирования, для оценки эффекта миссенс-замен использовали специализированные алгоритмы-предикторы эффекта генных вариантов. Сравнительный анализ спектра выявленных аминокислотных замен в исследованных группах показал, что во всех трех группах около 40 % индивидов были носителями «приватных» миссенс-вариантов в мтДНК, однако среди них в группе пациентов с нарушениями сердечного ритма чаще встречались варианты, классифицируемые предиктором APOGEE2 как «варианты неопределенного значения» (VUS), по сравнению с контрольной группой, в которой «приватных» миссенс-замен категории VUS не обнаружено (p = 0.0063 для точного критерия Фишера). Кроме того, группы различались по значениям phred-ранжированных значений CADD (Combined Annotation Dependent Depletion), которые были ниже для индивидов из контрольной группы. Полученные результаты указывают на то, что редкие варианты мтДНК могут вносить вклад в предрасположенность к сердечно-сосудистым заболеваниям, в частности в риск развития желудочковой тахикардии у некоторых пациентов.

## Introduction

Human mitochondrial DNA (mtDNA) exhibits a high degree of polymorphism, and, consequently, the proteins encoded by mitochondrial genes are similarly polymorphic. These proteins play a critical role in energy metabolism as essential components of the mitochondrial respiratory chain complexes. With the continuous growth of the human population, the burden of rare, so-called "private"genetic variants has increased substantially (Gao, Keinan, 2014), raising the likelihood that newly emerging gene variants – including mtDNA missense substitutions – may persist in the population even if they exert a mildly deleterious effect. While such variants are insufficient to cause severe hereditary disorders, they may contribute to the risk of common polygenic diseases.

The myocardium is one of the most energy-demanding tissues in the body. Most cardiovascular continuum disorders arise from myocardial ischemia, which is characterized by hypoxia, mitochondrial dysfunction, and oxidative stress (Kibel et al., 2020; Severino et al., 2020; Yang et al., 2022). Mitochondrial dysfunction, in turn, can exert an arrhythmogenic effect both through impaired ATP synthesis and via oxidative stress-induced membrane depolarization (Montaigne, Pentiah, 2015; Gambardella et al., 2017; van Opbergen et al., 2019). This is consistent with the frequent occurrence of cardiac arrhythmias in patients with mitochondrial diseases caused by pathogenic mtDNA mutations or nuclear gene defects affecting mitochondrial function (Ng, Turnbull, 2016). Conversely, severe cardiac arrhythmias – particularly paroxysmal ventricular tachycardia – are associated with a high risk of sudden cardiac death (Koplan, Stevenson, 2009; Chao et al., 2017), underscoring the importance of identifying hereditary risk factors for these conditions.Early research on mtDNA variants associated with cardiovascular disease risk primarily focused on common population variants and their combinations (haplogroups) (Palacín et al., 2011; Hudson et al., 2014; Golubenko et al., 2015, 2021; Kytövuori et al., 2020; Roselló-Díez et al., 2021). Advances in sequencing technologies now enable fast comprehensive analysis of the mitochondrial genome, leading to growing interest in the role of rare mtDNA variants in disease pathogenesis (Govindaraj et al., 2014, 2019; Hagen et al., 2015; Piotrowska-Nowak et al., 2019).The aim of this work was to study mtDNA rare missense variants in patients with ventricular tachycardia in comparison with patients without ventricular tachycardia and with relatively healthy individuals.

## Materials and methods

There were three groups of participants in the study. The "main" group consisted of patients hospitalized in the Department of Surgical Treatment of Complex Heart Rhythm Disorders and Electrical Pacing at the Cardiology Research Institute of Tomsk National Research Medical Center. All patients underwent implantation of a cardioverter-defibrillator (ICD) due to a history of ventricular tachycardia (VT) episodes, as part of primary or secondary prevention of sudden cardiac death (Bockeriaet al., 2017). The group included 127 individuals. Medical histories and diagnostic data were analyzed for all patients. Patients with severe comorbidities (cancer, NYHA class IV heart failure, or chronic kidney disease stages IV–V) were excluded. The majority were male (74.8 %), with a median age of 64.0 years (IQR: 59.0–71.0).

The "comparison" group (n = 53) comprised patients with stable ischemic heart disease and no his tory of myocardial infarction, VT, or indications for ICD implantation. Their median age was 67.0 years (IQR: 63.0–71.0). Clinical characteristics of all patients are provided in Table 1.In addition to the two groups of patients, a "control" group (n = 58) was formed, which consisted of Tomsk city residents who had no history of cardiovascular symptoms, including absence of heart rhythm disturbances; in addition, these individuals either had no stenosis of the carotid arteries, or the stenosis did not exceed 30 % (estimated by the ultrasound examination). The median age in this sample was 69.0 (62.0; 73.0) years, the ratio of men to women was 40:28 (69 % men).

**Table 1.**
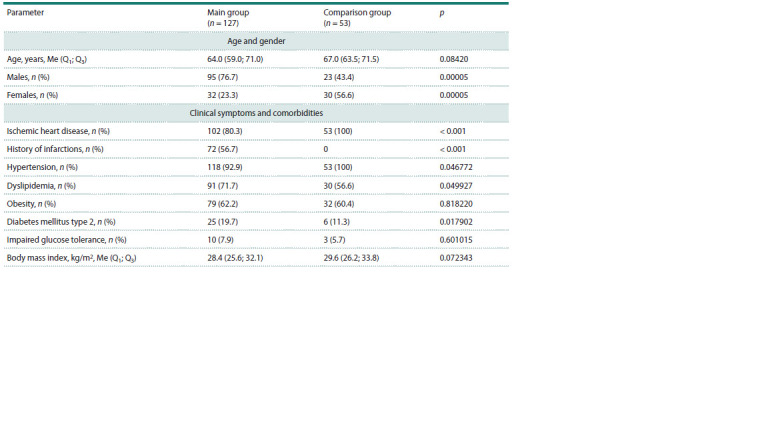
Clinical characteristics of the patients Note. p – significance level when comparing groups using the Pearson χ2 test (for frequencies) or the Mann–Whitney U-test (for quantitative characteristics).

Informed consent for participation in the study was obtained from all individuals included in the studied groups. The study protocol was approved by the biomedical ethics committees of the Research Institute of Medical Genetics and the Research Institute of Cardiology of the Tomsk National Research Medical Center.Venous blood samples (6–10 mL, EDTA) were collected, and DNA was isolated using phenol-chloroform extraction.

The complete mitochondrial genome was sequenced via high-throughput sequencing (next-generation sequencing, NGS). Mitochondrial DNA was amplified by long-range PCR with two overlapping fragments: 1) 9,065 bp (primers: 9397-9416 and 1892-1873 of the human mtDNA reference sequence) and 2) 11,170 bp (primers: 15195-15214 and 9796-9777). Overlapping regions spanned 9397-9796 and 15195-1873PCR was performed using the BioMaster LR HS-PCR (2x) kit (BioLabMix, Russia). PCR product concentration was quantified via Qubit (Thermo Fisher Scientific, USA) with Spectra Q BR reagents (Raissol, Russia). Equimolar pools of both PCR products (20 ng/μL) were prepared for each sample. DNA libraries were prepared using DNA library preparation kits designed for working with genomic DNA, with double indexing of the libraries. In particular, DNA Prep kits (Illumina, USA) and SG GM Plus kits (Raissol, Russia) were used. The manufacturer’s protocols were followed without modifications.

Sequencing was performed either on the MiSeq sequencer (Illumina, USA) using MiSeq reagent v.2 kit, 300 cycles, or on the GenoLab M sequencer (GeneMind, China) using GenoLab M V2.0 FCM reagent kit, 150 cycles.

After the data demultiplexing, fastq nucleotide reads were aligned to the reference human genome sequence (hg38) using DRAGEN 3.9.5 software, DNA pipeline (Illumina, USA). The resulting bam files were analyzed with mtDNA-specific software MtDNA-Server 2 (Weissensteiner et al., 2024). As a result, a list of nucleotide substitutions in comparison with the human mtDNA reference sequence (Andrews et al., 1999) was obtained, and an assessment of the mtDNA haplogroup for the identified haplotype was done according to the generally accepted human mtDNA tree (van Oven, Kayser, 2008). The mtDNA sequences in the *.fasta or *.txt format were also analyzed in the mtPhyl program (Eltsov, Volodko, 2011), which draws the phylogeny of the analyzed sequences and provides a list of missense variants divided into "haplogroup associated" and "private" substitutions, accompanied with amino acid conservation index for these substitutions.

To assess the effect of missense substitutions in mtDNA genes, we used the APOGEE 2 meta-predictor, which was developed specifically for mitochondrial DNA (Bianco et al., 2023), and in addition, CADD scores (Rentzsch et al., 2021) were analyzed. Data on these and other tools for assessing mtDNA missense substitutions are available online at the MitImpact project address: http://bioinformatics.css-mendel.it/ (Castellana et al., 2015). Statistical analysis was performed in JASP 0.19.3 (JASP Team, 2024). Group comparisons used Pearson’s χ² test (frequencies) or the Mann–Whitney U-test (quantitative variables).

## Results

The mtDNA sequencing results demonstrated high mitochondrial genome diversity in the studied cohorts, with nearly all individuals exhibiting unique mtDNA haplotypes. Only two haplotypes were observed twice, both occurring in the "main" patient group. The frequencies of major mtDNA haplogroups were distributed as follows: haplogroup H occurred at 34 % in the "main" group, 34 % in the "comparison" group, and 41 % in controls; haplogroup J, at 8, 9, and 14 %; haplogroup T, at 12, 9, and 3 %; and haplogroup U, at 30, 34, and 34 % respectively. These frequencies corresponded to the reported Tomsk population data (39 % for H, 7 % for J, 10 % for T, and 25 % for U) (Golubenko et al., 2021). Although trends suggested reduced haplogroup T and elevated haplogroup J frequencies in controls, along with increased haplogroup U frequency both in controls and in "comparison" patients, these differences did not reach statistical significance.

In the "main" patient group, we identified 61 private missense variants and 85 haplogroup-associated missense variants (Table 2). Altogether, 50 individuals (39 % of the group) carried private missense substitutions, including 7 patients with two variants and 2 patients with three variants. The "comparison" group exhibited 28 private missense variants (found in 23 individuals, 43 % of this group, including 5 carriers of two variants) compared to 45 haplogroup-associated variants. The control group showed 35 private missense variants distributed among 23 individuals (40 % of controls), with 8 individuals harboring two variants and 2 individuals carrying three variants.

**Table 2.**
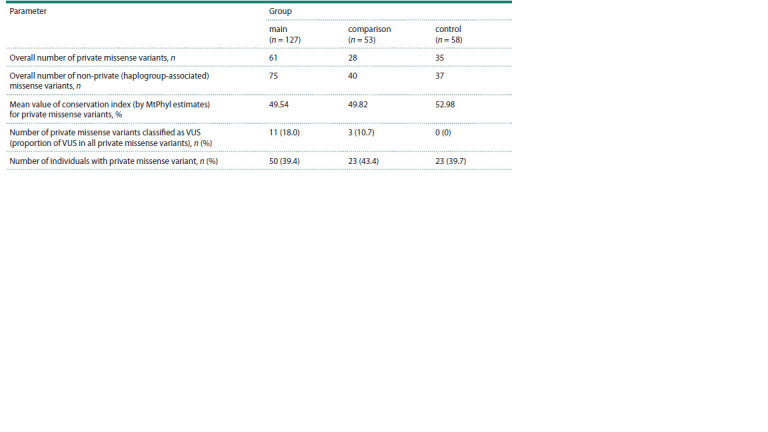
Characteristics of mtDNA missense polymorphism in the studied groups

Elson’s neutrality test (Elson et al., 2004) revealed no statistically significant deviations in the ratio of synonymous to non-synonymous substitutions from neutral selection expectations across groups. Similarly, we observed no significant differences in mean amino acid conservation indices between private and haplogroupassociated variants.

**Fig. 1.**
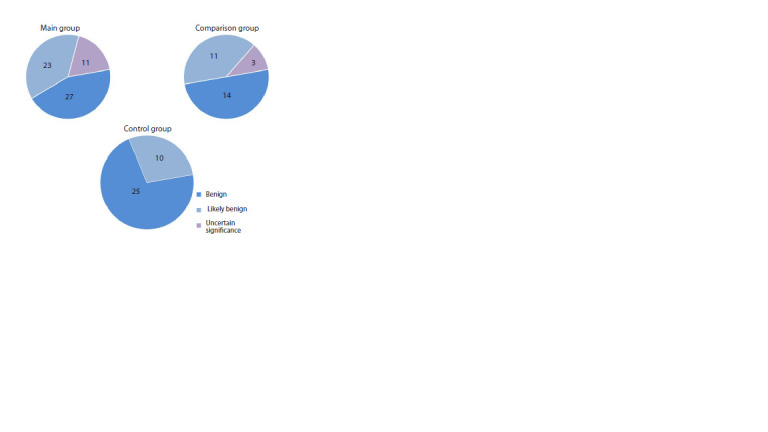
Attribution of private mtDNA missense variants to the different categories of pathogenicity (the numbers indicate the number of variants in the corresponding category).

The APOGEE2 meta-predictor classifies missense variants using the standard five-tier pathogenicity system (benign, likely benign, variants of uncertain significance (VUS), likely pathogenic, and pathogenic) (McCormick et al., 2020). Variants with APOGEE2 scores of 0.265–0.716 are categorized as VUS, while higher and lower scores indicate likely pathogenic/pathogenic and likely benign/benign variants, respectively (Bianco et al., 2023). No private missense variants in our cohorts met criteria for pathogenic or likely pathogenic. In contrast, the main group contained 11 private VUS variants (18 % of its private variants), while the comparison group had three (10.7 %), and controls showed none (Table 2). This represents a significant accumulation of non-benign private variants in the main group compared to controls (p = 0.0063, Fisher’s exact test). Differences between other group pairings were non-significant. The distribution of private variants across pathogenicity categories is illustrated in Figure 1.

The complete list of private missense substitutions classified as VUS is presented in Table 3. Notably, two variants (T3394C and G13708A), though identified as Main group 23 27 11 11 10 25 14 3 Control group Benign Likely benign Uncertain significance Comparison group Fig. 1. Attribution of private mtDNA missense variants to the different categories of pathogenicity (the numbers indicate the number of variants in the corresponding category). Table 3. Private mtDNA missense variants classified as VUS (APOGEE2) No. mtDNA change Gene Amino acid change APOGEE2 score Patient group mtDNA haplogroup 1 T3394C MT-ND1 Y30H 0.5822 Main J1b1a1 2 C6489A MT-CO1 L196I 0.3289 Main T 3 G6510A MT-CO1 A203T 0.2836 Comparison H6a1a 4 C8369T MT-ATP8 P2S 0.2767 Main U5a2a1b 5 T9205C MT-ATP6 227Q – Main J1a1b1 6 G9738A MT-CO3 A178T 0.3554 Main R2 7 T10237C MT-ND3 I60T 0.6661 Main HV 8 G11696A MT-ND4 V313I 0.3383 Main K1 9 T12075C MT-ND4 M439T 0.3560 Main U5a1b 10 C13036T MT-ND5 P234S 0.4992 Main K1b1 11 G13708A MT-ND5 A458T 0.3070 Main T1a 12 T14291A MT-ND6 E128V 0.4046 Main H36 13 A14841G MT-CYTB N32S 0.2743 Comparison H1j8 14 G15152A MT-CYTB G136S 0.2924 Comparison U5a1 private in our patients, appear in multiple haplogroups on the human mtDNA phylogenetic tree – particularly G13708A, which characterizes West-Eurasian haplogroup J. Another private variant resulted not in an amino acid substitution but in replacement of a stop codon with glutamine (T9205C, MT-ATP6 Ter227Gln) in the ATP6 gene. While APOGEE2 cannot score such variants, ClinVar database classifies it as VUS (https:// www.ncbi.nlm.nih.gov/clinvar/variation/693124/, accessed 24.02.2025). Similarly, a stop-to-lysine variant (A7444G, MT-COI Ter514Lys), associated with haplogroup V7 and found in the main group, was previously considered pathogenic due to protein elongation but has been reclassified as "likely benign" after having been reviewed by ClinVar experts (https://www.ncbi.nlm. nih.gov/clinvar/variation/9663/, accessed 24.02.2025).

**Table 3.**
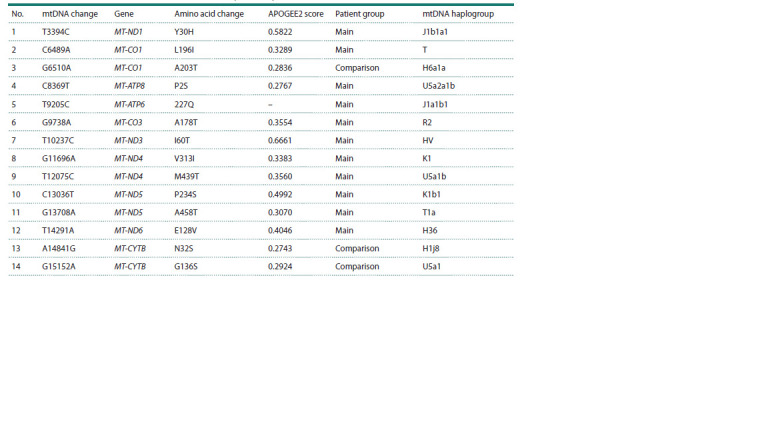
Private mtDNA missense variants classified as VUS (APOGEE2)

Of all VUS, 50 % are located in the genes encoding subunits of the first complex of the respiratory chain (NADH dehydrogenase), which is consistent with the total length of these genes, which encompass 65 % of the total length of all protein-coding mtDNA genes. It is interesting, however, that all three VUS identified among the patients of the comparison group were located not in the NADH dehydrogenase genes but in the cytochrome b gene (two variants) and cytochrome c oxidase gene (one variant). It can also be noted that while haplogroup H is the most common among Europeans (about 40 % of the population), only three VUS, i. e. 21 %, belonged to this haplogroup (H6a1a, H36, H1j8), whereas a significant portion of VUS (36 %) belonged to haplogroup U (U5a2a1b, U5a1b, K1, K1b1, U5a1), and another 36 % belonged to the R2’JT cluster (J1b1a1, T, J1a1v1, R2, T1a). It might be assumed that the appearance of VUS on the background of haplogroup R2’JT may be a risk factor for the development of arrhythmia, including VT, but the total number of VUS in our study is too small to perform any statistical tests, so this issue requires additional research.

The classification of variants into pathogenicity classes is "categorical"; however, it relies on quantitative scales of the effect estimates. One of these estimates is CADD (combined annotation dependent depletion), an integrated metric based on machine learning, which uses more than 60 tools for annotating all possible genetic variants, followed by calculating the probability of their appearance in the genome and ranking all possible variants according to this probability. The logarithm of this score (phred-like ranking) is used to identify the least "probable" variants in the genome, which are therefore the variants with the greatest effect. According to the developers’ recommendation, the minimum (threshold) value of the CADD phred score for considering a possibility of functional significance is 10. It means that the variant is among the 10 % most significant of all theoretically possible variants in the genome (Rentzsch et al., 2021).

**Fig. 2.**
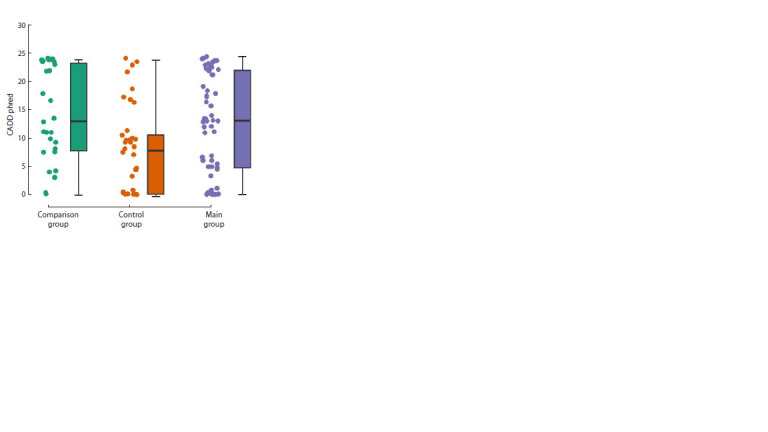
Plot of CADD phred scores for all private missense substitutions in the studied groups.

The plot of CADD phred score values for all "private" missense variants is shown in Figure 2. In all groups, there were missense substitutions with this parameter value greater than 10; however, in the control group, only 26.6 % of "private" missense substitutions were in this zone, and the median value of this parameter was 8.3, while in the patient groups, the median value was 13 (main group) and 13.2 (comparison group). In total, 61.7 % of private substitutions in the main group and 64.3 % in the comparison group had a CADD phred score greater than 10. This differentiation was statistically significant both according to the results of variance analysis (p = 0.014) and according to the nonparametric Kruskal–Wallis criterion (p = 0.011).

## Discussion

Unlike evolutionarily "established" combinations of mtDNA variants designated as haplogroups, all newly emerging variants are "private", meaning they are present only in the examined individual and probably in his/her close maternal relatives. When the population size constantly increases, there is an excess of "private" gene variants in the population (Gao, Keinan, 2014). Such newly arising mtDNA variants may influence the phenotype. When the variant has a strong negative effect on the phenotype, it may be eliminated from the population by natural selection; however, if the effect is small, the variant can persist in the population for many generations and even spread due to genetic drift.

Assessing the effect of a missense substitution in a gene on the structure and function of the encoded protein is an important issue. Despite the variety of algorithms and predictors developed for in silico effect estimation, the results of these studies do not always correspond to the true effect of specific missense substitutions. This is partly due to the insufficient experimental data on the pathogenicity of various variants, since only a small share of all possible amino acid substitutions has been studied in this regard, so extrapolation of these patterns to the entire data set is not always correct. In addition, epistatic interactions of amino acid residues between amino acid residues within or between protein subunits may contribute to effect variability, where additional amino acid substitutions could compensate for or exacerbate the effect of the analyzed substitution.

APOGEE2 is a meta-predictor that uses for its assessment evolutionary conservation; protein structural characteristics, including tertiary structure data and Gibbs free energy change (ΔΔG); effect estimates obtained from various predictor programs: PolyPhen2, SIFT, Fathmm, PROVEAN, MutationAssessor, EFIN, CADD, PANTHER, PhDSNP, SNAP, and MutationTaster2 (Bianco et al., 2023). This tool has the highest sensitivity (87 %) and specificity (90 %) compared to other predictors, according to the paper.

Distribution of values from the quantitative predictor of functional significance (CADD) showed that private mtDNA missense substitutions in the control group were characterized on average by lower values of this paraComparison of the fraction of variants with uncertain significance (VUS) among private missense substitutions showed a higher proportion of VUS in the main patient group versus controls. At the same time, missense substitutions of the VUS category were also registered in the comparison group patients, though their APOGEE2 scores were minimal (Table 3). The ratio of such variants to the total number of private missense substitutions in this sample (3/28, or 10.7 %), while lower than in the main group (11/61, or 18 %), showed no statistically significant difference (p > 0.05 by Fisher’s exact test). Thus, the high frequency of VUS-category missense substitutions may be associated not with arrhythmia risk specifically but with predisposition to cardiovascular diseases in general. Some previous mtDNA studies have also identified rare and private substitutions, including missense variants, which can be classified as VUS or even as likely pathogenic variants – for example, in patients with hypertrophic cardiomyopathy (Govindaraj et al., 2014; Hagen et al., 2015), dilated cardiomyopathy (Govindaraj et al., 2019), and atherosclerosis (Piotrowska- Nowak et al., 2019).meter, and these differences were statistically significant. Similar to the proportion of VUS, the two groups of patients (the main and comparison groups) did not differ from each other in mean CADD values.

Notably, two identified variants classified as VUS may be characteristic of certain mtDNA haplogroups. The G13708A substitution is one of the defining variants for haplogroup J, which is known to enhance expressivity of the pathogenic G11778A variant causing Leber’s optic atrophy in European populations (Torroni et al., 1997). The T3394C substitution similarly enhances manifestation of pathogenic variant G11778A but in Asian populations (Ji et al., 2019). Both substitutions occur repeatedly in human mtDNA phylogeny (www. phylotree.org). Remarkably, in our study, the patient with private T3394C substitution had mtDNA belonging to haplogroup J (specifically J1b1a1, Table 3), meaning they also carried the G13708A substitution. Thus, this individual had two missense substitutions, each representing an unfavorable "background" promoting manifestation of pathogenic mtDNA variants. In this regard, it is interesting that, according to the published data, similar combinations of mtDNA variants were identified in Parkinson’s disease, where variants typically associated with certain haplogroups ("out of place" variants) were more frequent in patients than in controls (Müller-Nedebock et al., 2022). Leber optic atrophy and Parkinson’s disease are not cardiovascular diseases, but these examples may present general patterns of mtDNA variants effects manifestation.

Classifying a genetic variant as a VUS does not necessarily indicate a negative effect – it indicates only a higher probability that such a variant somehow influences the phenotype, hence the term "variant of uncertain significance". Nevertheless, the excess of such variants in the group of patients with life-threatening cardiac arrhythmias (ventricular tachycardia) and a high risk of sudden death revealed in our study suggests that, at least in some cases, the risk of sudden death may be increased by rare mtDNA variants with a negative effect, resulting in a decrease in the mitochondrial function. It can be assumed that, under normal conditions, minor deviations from optimal function of mitochondrial protein complexes may be compensated by increased mitochondrial gene expression, mitochondrial biogenesis, or modulation of specific biochemical pathways. Under cellular stress condition, however, this "borderline" mitochondrial dysfunction may become critical for the myocardial pathology development.

Whether such variants represent an arrhythmiaspecific risk factor or generally increase cardiovascular disease risk remains an open question. Further studies are required in patient cohorts with diverse cardiovascular pathologies. Assessing genetic background effects on rare missense substitutions (potential epistatic interactions) will require larger samples, as private VUScategory missense variants occur in fewer than 10 % of patients. In addition, it should be noted that our study did not consider heteroplasmy – a situation when only a portion of the mtDNAs have the variant, which can be either inherited or somatically arisen de novo. Due to the lack of the possibility of analyzing the DNA of parents (mothers), we could not assess de novo variant occurrence. All mtDNA variants described here were homoplasmic.

## Conclusion

Comparative analysis of rare (private) missense substitution spectra in mtDNA protein-coding genes among cardiovascular disease patients – particularly those with life-threatening arrhythmias – revealed several missense substitutions that may be classified as VUS, suggesting possible functional impacts on mitochondrial respiratory chain proteins. No such variants were found in the control group of individuals without clinical cardiovascular symptoms.

Groups showed no differences in overall mtDNA missense polymorphism characteristics (the total number of missense substitutions, the proportion of carriers of private missense variants in the group, more than one private missense substitution in one individual, the amino acid conservation index). However, there were statistically significant differences between the main group (with a history of ventricular tachycardia and a high risk of sudden death) and the control group in the proportion of VUS among private missense variants. In addition, differences between the groups were revealed for the values of the quantitative score characterizing the possibility of the functional significance of variants (CADD score). These results allow us to assume that it is rare missense substitutions of mtDNA that may have functional impact and contribute to the predisposition to the cardiovascular continuum diseases, including the development of ventricular tachycardia in patients.

## Conflict of interest

The authors declare no conflict of interest.
